# B cell-dependent EAE induces visual deficits in the mouse with similarities to human autoimmune demyelinating diseases

**DOI:** 10.1186/s12974-022-02416-y

**Published:** 2022-02-23

**Authors:** Sandrine Joly, Julius Baya Mdzomba, Léa Rodriguez, Françoise Morin, Luc Vallières, Vincent Pernet

**Affiliations:** 1 Department of Ophthalmology, Inselspital, Bern University Hospital, University of Bern, Bern, Switzerland; 2 Regenerative Medicine Unit, University Hospital Center of Quebec, Laval University, Quebec City, QC Canada; 3 Department of Molecular Medicine, Faculty of Medicine, Laval University, Quebec City, QC Canada; 4 Neuroscience Unit, University Hospital Center of Quebec, Laval University, Quebec City, QC Canada; 5 Center for Experimental Neurology (ZEN), University of Bern, Bern, Switzerland; 6 Department of Neurology, Inselspital, Bern University Hospital, University of Bern, Sahli Haus 1, UG Büro 1, Freiburgstrasse 14, 3010 Bern, Switzerland

**Keywords:** Multiple sclerosis, Experimental autoimmune encephalomyelitis, Electroretinogram, Optic neuritis, B cells, Inflammation

## Abstract

**Background:**

In the field of autoimmune demyelinating diseases, visual impairments have extensively been studied using the experimental autoimmune encephalomyelitis (EAE) mouse model, which is classically induced by immunization with myelin oligodendrocyte glycoprotein peptide (MOG_35–55_). However, this model does not involve B cells like its human analogs. New antigens have thus been developed to induce a B cell-dependent form of EAE that better mimics human diseases.

**Methods:**

The present study aimed to characterize the visual symptoms of EAE induced with such an antigen called bMOG. After the induction of EAE with bMOG in C57BL/6J mice, visual function changes were studied by electroretinography and optomotor acuity tests. Motor deficits were assessed in parallel with a standard clinical scoring method. Histological examinations and Western blot analyses allowed to follow retinal neuron survival, gliosis, microglia activation, opsin photopigment expression in photoreceptors and optic nerve demyelination. Disease effects on retinal gene expression were established by RNA sequencing.

**Results:**

We observed that bMOG EAE mice exhibited persistent loss of visual acuity, despite partial recovery of electroretinogram and motor functions. This loss was likely due to retinal inflammation, gliosis and synaptic impairments, as evidenced by histological and transcriptomic data. Further analysis suggests that the M-cone photoreceptor pathway was also affected.

**Conclusion:**

Therefore, by documenting visual changes induced by bMOG and showing similarities to those seen in diseases such as multiple sclerosis and neuromyelitis optica, this study offers a new approach to test protective or restorative ophthalmic treatments.

**Supplementary Information:**

The online version contains supplementary material available at 10.1186/s12974-022-02416-y.

## Introduction

Multiple sclerosis (MS) is a prevalent demyelinating autoimmune disease of the central nervous system (CNS) [1]. While many treatments allow to decrease peripheral activation and CNS infiltration of autoreactive lymphocytes [2], treatments stimulating myelin or axon regeneration are not yet available. Potential targets are Nogo-A and its receptor subunits, especially leucine-rich repeat and immunoglobulin-like domain-containing protein 1 (Lingo-1) [3, 4]. Indeed, Lingo-1-neutralizing antibodies have been shown to enhance functional recovery and preserve axonal integrity in EAE [5, 6]. However, a clinical trial with the anti-Lingo-1 antibody opicinumab failed in patients with relapsing–remitting MS [7]. The gap between preclinical and clinical observations may be attributed in part to the use of an experimental model that imperfectly reproduces the pathophysiological mechanisms of MS [8]. In particular, the fact that classical EAE induced with peptides such as MOG_35–55_ does not depend on pathogenic B cell responses, such as antigen presentation to T cells and antibody secretion [9], is a major difference with MS [10–12]. It is now possible to take these responses into consideration by inducing a B cell-dependent form of EAE with antigens such as the extracellular domain of MOG (MOG_1–125_) of different species. In the present study, we sought to characterize visual changes in such a model using the novel antigen bMOG [10], which is mouse MOG_1–125_ with a single mutation (S42P) abolishing the immunodominant T cell epitope (residues 35–55).

In MS, vision is impaired in ~ 70% of cases [13]. Loss in visual acuity, contrast sensitivity and color vision result from optic neuritis [1, 14] and the death of retinal ganglion cells (RGCs), the output neurons of the retina [1, 15]. In MOG_35–55_ EAE, some important features of MS are observed such as RGC death, that can occur before the onset of motor symptoms [16–19] or during the chronic phase following retrograde axonal degeneration [20]. In addition, electroretinogram (ERG) recordings suggest that the function of retinal cells upstream of RGCs, in layers lacking myelin, may also be affected in EAE [21, 22] and MS [23–25]. However, the reported ERG function changes are not always consistent in EAE [16, 17], MS [26] and optic neuritis [27]. We therefore aimed to record ERGs at different stages of bMOG EAE in order to clarify its evolution over time and its relevance for monitoring disease progression.

Here, we report a persistent decrease in visual acuity in bMOG EAE mice, although motor symptoms improved over time. ERG recordings reveal a marked, but transient decrease in the activity of outer retinal cell layers, including those of photoreceptors and bipolar cells. In addition, photopic ERGs and photopigment expression analyses suggest that the pathway of M-cones, that are photoreceptors sensitive to green light in daylight conditions, is particularly affected. ERG impairments in bMOG mice are associated with inflammation and share similarities with human demyelinating autoimmune diseases.

## Materials and methods

### Mice

Adult C57BL/6J male mice were purchased from Jackson Laboratory and were used at 2–2.5 months of age. Animal experiments were carried out in accordance with the guidelines of the Canadian Council on Animal Care and of the Laval University Animal Welfare Committee.

### Experimental autoimmune encephalomyelitis induction

Mice were subcutaneously injected with a total of 200 µl of emulsion containing 500 μg of bMOG [10] dissolved in saline and mixed with an equal volume of complete Freund’s adjuvant (CFA; Millipore-Sigma, Oakville, ON, Canada) supplemented with 500 μg of killed *Mycobacterium tuberculosis* H37 RA (Difco Laboratories). Mice received an intraperitoneal injection of 20 μg/kg of pertussis toxin (List Biological Laboratories, Campbell, CA, USA) immediately and 2 days after bMOG immunization. Mice were weighed and scored daily as follows: 0 = no visual sign of disease; 0.5 = partial tail paralysis; 1 = complete tail paralysis; 1.5 = weakness in one hind limb; 2, weakness in both hind limbs; 2.5 = partial hind limb paralysis; 3 = complete hind limb paralysis; 3.5 = partial forelimb paralysis; 4 = complete forelimb paralysis; 5 = dead or killed for human reasons. Cumulative EAE scores were calculated by summing daily scores for 15 days.

### Optokinetic reflex (OKR) test for visual acuity assessment

To evaluate the visual acuity of mice, the optokinetic reflex was tested using the OptoDrum system (Striatech GmbH Vor dem Kreuzberg, Tübingen, Germany). Freely moving mice were placed on a platform in the middle of an arena surrounded by four computer screens showing moving gratings at variable spatial frequencies [28–30]. The tracking movement of the neck in the temporal-to-nasal direction was monitored to determine the spatial frequency threshold (in cycles/degree) of the optokinetic reflex. The function of each eye was separately evaluated by changing the direction of the visual stimulus.

### Electroretinogram recording

A Ganzfeld system (Phoenix Research Labs, Pleasanton, CA, USA) was used to record scotopic (dark-adapted) and photopic (light-adapted) ERGs in mice anesthetized with ketamine/xylazine (10:1 mg/kg), as before [31]. Recordings were performed 16 and 32 days after immunization. For scotopic recordings, mice were adapted to complete darkness for ~ 12 h the day before. Prior to recording, pupil was dilated with a drop of 1% mydriacyl tropicamide applied on the cornea. A sterile ophthalmic gel (Tear-Gel, Bausch & Lomb) was used to prevent corneal desiccation and to allow electrical conductance between the cornea and the electrode (gold-plate objective lens). Scotopic full-field ERGs (bandwidth: 2–1000 Hz) were obtained in response to light stimulation with increasing flash intensities, ranging from − 1.7 to 2.2 log cd.s.m^−2^ (inter-stimulus interval, 20 ms; flash duration, 1 ms; 0.3 log-unit increment). Photopic ERG responses were induced by flash stimulations of intensity ranging from 1.0 to 2.8 log cd.s.m^−2^ (inter-stimulus interval, 20 ms; flash duration, 1 ms; average of 20 flashes, 0.6 log-unit increment). The use of green (504 nm) and UV (365 nm) lights allowed to record the M-cone- and S-cone-dependent ERG waveforms, respectively. The amplitude of the b-wave was measured from the a-wave trough to the highest peak. Implicit times (latencies) were calculated from flash onset to peak.

### Tissue preparations for histology

Mice were euthanized using ketamine/xylazine (90:10 mg/kg). Tissues were fixed by intracardial perfusion of phosphate-buffered saline (PBS) and 4% paraformaldehyde (PFA). Eyes and optic nerves were dissected for retinal and optic nerve sections (14-μm thick) or retinal flat-mount preparations.

### Retinal ganglion cell and cone photoreceptor survival analysis

The survival of RGCs was evaluated on retinal flatmounts 18 and 35 days after immunization. Retinal flatmounts were post-fixed overnight in 4% PFA, rinsed with PBS three times, incubated for 1 h in a blocking solution (PBS containing 5% bovine serum albumin and 0.3% Triton X-100) and incubated for 5 days at room temperature with guinea-pig anti-RNA binding protein with multiple splicing (RBPMS) (Table 1). After intensive washing, retinal flatmounts were incubated for 2 days at room temperature with appropriate secondary antibody diluted in blocking solution. Vectashield (BioLynx, Brockville, ON, Canada) was used as antifade mounting medium. Cells were counted in regions of 62,500 μm^2^ at 0.5, 1, 1.5, and 2 mm from the optic disk in the four retinal quadrants. The density of S- and M-cones was also quantified on retinal flatmounts 18 days after immunization using S-opsin and M-opsin specific antibodies (Table 1).

**Table 1:** Antibodies used for immunofluorescence (IF) and western blotting (WB)

Name	Species	Dilution IF	Dilution WB	Source
Olig2	Rabbit	1:500		Millipore
FluoroMyelin	N/A	1:300		Life Technologies
RBPMS	Guinea pig	1:500		PhosphoSolutions
M-opsin	Rabbit	1:500	1:5000	Millipore
S-opsin	Goat	1:200	1:2000	Santa Cruz
Peanut agglutinin	N/A	1:250		Vector Laboratories
GAPDH	Mouse		1:20,000	Abcam
GFAP	Rabbit	1:500		Dako
GS	Mouse	1:1000		Millipore
Iba-1	Goat	1:500		Novus Biologicals
CD68	Rat	1:1000		Abcam
Syt2	Mouse	1:200		Abcam
ChAT	Goat	1:100		Millipore
NF-H	Mouse	1:500		Millipore
Melanopsin	Rabbit	1:2500		Advanced Targeting Systems
B3T	Mouse	1:1000		Promega
P-Erk_1/2_	Rabbit	1:100		Cell Signaling

*N/A* not applicable

### Immunofluorescence on retinal and optic nerve cryosections

Retinal eye cups and optic nerves were post-fixed overnight in 4% PFA and immersed in a solution of 30% sucrose for cryoprotection before tissue embedding in Optimal Cutting Temperature medium (Cedarlane, Burlington, ON, Canada). Fourteen-μm-thick sections were collected on Superfrost microscope glass slides. For immunostaining, slices were incubated for 1 h in a blocking solution and then overnight with primary antibodies at 4 °C (Table 1). After three washes with PBS, sections were incubated at room temperature with the appropriate secondary antibodies. Slides were mounted with Vectashield. For microscopy and image acquisition, mosaic pictures were taken with a Zeiss AxioImager M2 microscope equipped with a motorized platform and the ZEN software or with a Zeiss LSM 700 scanning confocal microscope. All quantifications were performed using 5–6 central retinal or optic nerve cuts/mouse. Demyelination was studied in the optic nerve by incubating longitudinal slices for 20 min with FluoroMyelin (1:300) at room temperature. Oligodendrocyte nuclei were labeled using rabbit anti-oligodendrocyte transcription factor 2 (Olig2) antibody (Table 1). FluoroMyelin staining was quantified by densitometry with the ImageJ (NIH) software. The number of Olig2^+^ cells was quantified on complete longitudinal sections of optic nerves.

### Western blot analysis

Retinae were quickly isolated from the eyes, snap frozen in liquid nitrogen and stored at − 80 °C until protein lysate preparation. Retinae were homogenized for 60 min on ice in Eppendorf tubes containing lysis buffer (20 mM Tris–HCl, 0.5% CHAPS, pH 8.0) and protease/phosphatase inhibitor (Roche Diagnostics, Laval, QC, Canada) and centrifuged for 15 min at 15,000×*g* at 4 °C. Supernatants were then retrieved and used for protein assay (BioRad, Mississauga, ON, Canada). Retinal proteins (20 μg/well) were resolved by electrophoresis on 4–12% gradient polyacrylamide gels and transferred to nitrocellulose membranes. Nitrocellulose membranes were pre-incubated in a blocking solution of 5% bovine serum albumin dissolved in TBST (Tris–base 0.1 M, 0.2% Tween 20, pH 7.4) for 1 h at room temperature, incubated with primary antibodies overnight at 4 °C (see Table 1). After washes, membranes were incubated with the appropriate horseradish peroxidase-conjugated secondary antibodies. Chemiluminescent bands were detected with LiCor Western Sure Premium Chemiluminescent Substrate (Mandel, Guelph, ON, Canada) in a LiCor C-Digit blot scanner (Mandel).

### Real-time qPCR

Under deep anesthesia with isoflurane, mice were sacrificed by cervical dislocation. Retinae were rapidly dissected, flash-frozen in liquid nitrogen and stored at − 80 °C. RNA was isolated using the RNeasy Isolation kit (Qiagen, Toronto, ON, Canada). Residual genomic DNA was eliminated by DNase treatment (Qiagen). Oligo (dt) and M-MLV reverse transcriptase (Fisher Scientific, Toronto, ON, Canada) were used to transform equal amounts of RNA for reverse transcription. Amplification of 10 ng of cDNA with the SYBR Green I Master polymerase ready mix (Roche Diagnostics Canada) was done using the Light Cycler 480 thermocycler (Roche Diagnostics Canada). Primer pairs were designed to span the intronic sequences or to cover exon–intron boundaries (see Table 2 for sequences). The comparative threshold cycle (ΔΔCT) method was used to calculate the relative quantity. *Gapdh* was used to normalize cDNA levels. Each reaction was done in triplicate.

**Table 2:** Primer pair sequences

Gene names	Forward primer (5ʹ–3ʹ)	Reverse primer (5ʹ–3ʹ)
*Gapdh*	CAGCAATGCATCCTGCACC	TGGACTGTGGTCATGAGCCC
*Cd68*	ACCTACATCAGAGCCCGAGTACAG	TTCTGCGCCATGAATGTCCACTG
*Edn2*	AGACCTCCTCCGAAAGCTG	CTGGCTGTAGCTGGCAAAG
*Fgf2*	TGTGTCTATCAAGGGAGTGTGTGC	ACCAACTGGAGTATTTCCGTGACCG
*Gfap*	CCACCAAACTGGCTGATGTCTAC	TTCTCTCCAAATCCACACGAGC
*Gnat1*	GAGGATGCTGAGAAGGATGC	TGAATGTTGAGCGTGGTCAT
*Gnat2*	GCATCAGTGCTGAGGACAAA	CTAGGCACTCTTCGGGTGAG
*Opn1mw*	CTCTGCTACCTCCAAGTGTGG	AAGTATAGGGTCCCCAGCAGA
*Opn1sw*	TGTACATGGTCAACAATCGGA	ACACCATCTCCAGAATGCAAG
*Rho*	CAAGAATCCACTGGGAGATGA	GTGTGTGGGGACAGGAGACT
*Vim*	TACAGGAAGCTGCTGGAAGG	TGGGTGTCAACCAGAGGAA

### Total RNA sequencing

RNA quality was validated with a Bioanalyzer (TapeStation 2200; Agilent Technologies, Santa Clara, USA). The NEBNext Ultra II directional RNA library prep kit for Illumina (New England Biolabs Inc., Ipswich, MA, USA) was used to prepare total RNA sequencing libraries, according to the manufacturer’s instructions. Briefly, we used 300 ng of total RNA for EAE and control samples and 160 ng of total RNA for CFA samples, in which external RNA controls Ambion^®^ ERCC Spike-In Control Mixes (ThermoFisher, Canada) were added. Ribosomal RNA (rRNA) was removed using the RNaseH-based method (NEBNext rRNA depletion kit; New England Biolabs Inc., Ipswich, MA, USA). Following purification with Agencourt RNAClean XP beads (Beckman Coulter, Mississauga, Ontario, Canada), RNA was fragmented using divalent cations under elevated temperature, and then used as a template for cDNA synthesis by reverse transcriptase with random primers. The specificity of the strand was obtained by replacing dTTP with dUTP. The first strand cDNA was converted to double-stranded cDNA and end-repaired. Ligation of adaptors was followed by a purification step with the AxyPrep Mag PCR Clean-up kit (Axygen, Big Flats, NY, USA), by an excision of the strands containing the dUTPs, and finally by a PCR enrichment step of 10 cycles to incorporate specific indexed adapters for multiplexing. The quality of the final libraries was examined with a DNA Screen Tape D1000 on a TapeStation 2200 (Agilent Technologies, Santa Clara, USA) and its quantification was done on the QBit 3.0 fluorometer (ThermoFisher Scientific, Canada). Subsequently, libraries with unique index were pooled together in equimolar ratio and sequenced for paired-end 100-pb sequencing on a NovaSeq 6000 flowcell S2 at the Next-Generation Sequencing Platform, Genomics Center, CHU de Québec-Université Laval Research Center, Québec City, Canada. The average insert size for the libraries was 280 bp. The mean coverage/sample was 45 million of paired-end reads.

Reads were trimmed using fastp v0.20.0 [32]. Quality check was performed on raw and trimmed data to ensure the quality of the reads using FastQC v0.11.8 and MultiQC v1.8 [33]. Quantification was performed with Kallisto v0.46.2 [34] against the *Mus musculus* transcriptome (GRCm38 downloaded from Ensembl release 100). Principal component analysis was performed using the ade4 v1.7-17 R package. The ComplexHeatmap package v2.6.2 was used to create heatmaps [35]. All other graphical representations were produced with the ggplot2 v3.3.3 package. Differential expression analysis was performed using the DESeq2 v1.30.1 package [36]. All R analysis were done in R v4.0.3.

### Statistics

Statistical analyses were performed with the GraphPad Prism software (La Jolla, CA, USA). For OKR and ERG measurements, a two-way ANOVA followed by Tukey post hoc test was done. Similarly, RT-qPCR results were analyzed using one-way ANOVA followed by Tukey post hoc test. For cell counts and FluoroMyelin staining, statistical differences were evaluated using Student’s unpaired *t*-test or one-way ANOVA.

## Results

### Sustained visual deficits in B cell-dependent EAE

**Fig. 1:**
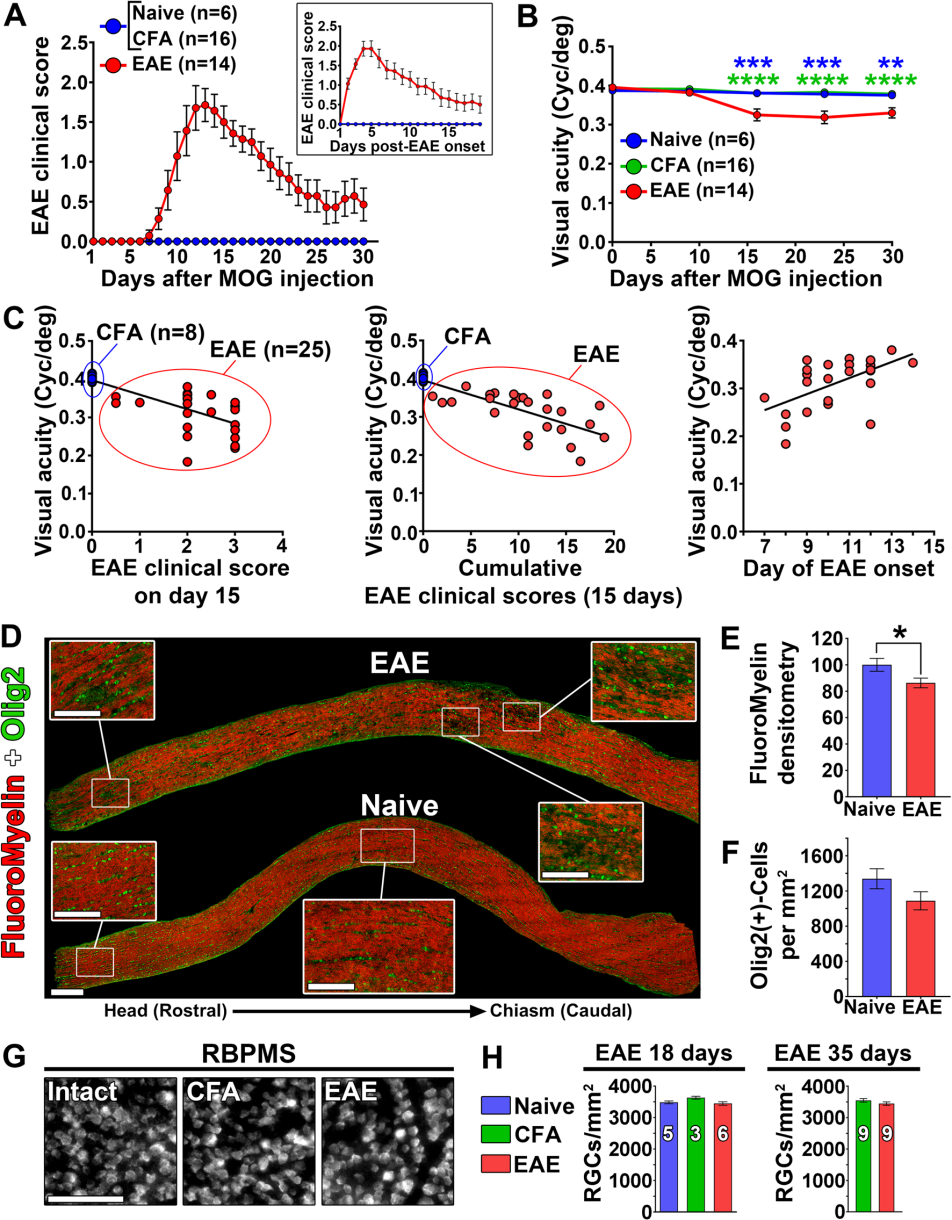
Visual function deficits are sustained in B cell-dependent experimental autoimmune encephalomyelitis. **A** Motor symptoms were daily assessed using a 0–5 clinical scoring scale after the injection of bMOG or CFA only. **B** For visual function assessment, optomotor visual acuity was followed in the OptoDrum system allowing spatial frequency sensitivity evaluation in awake mice. **C** Visual acuity loss was correlated with the clinical scores, cumulative clinical scores and onset of EAE at 15 days. **D** Optic nerve demyelination was evaluated by staining myelin with FluoroMyelin in cryosections. Oligodendrocytes were identified with Olig2 transcription factor. **E** Densitometric measurement of FluoroMyelin staining showed significant loss of myelin associated with EAE (*n* = 7) compared with control mice (*n* = 6). **F** The number of Olig2^+^ cells was not different between EAE and CFA-treated optic nerves. **G** Neuronal survival was examined on retinal flatmounts stained for RBPMS, a specific marker for RGCs. **H** On days 18 (active disease phase) and 35 (chronic phase), the density of RBPMS^+^ RGCs did not differ between the three experimental groups. Scale bars: **D** = 200 µm (left lower corner), 100 µm (close-ups); **G** = 100 µm. Statistics: **B** = two-way ANOVA, Tukey post hoc test; **E** = unpaired *t*-test; *: *P* < 0.05, **: *P* < 0.01, ***: *P* < 0.001, ****: *P* < 0.0001. Green stars indicate statistical significance between EAE and CFA-treated mice. Blue stars indicate statistical significance between EAE and naive mice

The loss of visual acuity has been shown to parallel motor deficits in classical EAE induced with MOG_35–55_ peptide [37–39]. To examine whether a similar loss occurs in B cell-dependent EAE induced with bMOG, we monitored mice for motor symptoms daily and for visual acuity at different time points (days 1, 9, 15, 23, 30) after bMOG injection. Motor symptoms started at day ~ 7 and peaked at day ~ 13 (Fig. 1A, Additional file 1: Fig. S1A). Thereafter, the symptoms gradually improved until day 30 (Fig. 1A), as previously observed [10]. In contrast, visual acuity decreased from day 15, i.e., after EAE onset, and remained lower than in controls until the end of the experiment on day 30 (Fig. 1B, Additional file 1: Fig. S1B). On day 15, the extent of visual loss correlated with cumulative clinical scores (Fig. 1C). These data show that B cell-dependent EAE induces a sustained loss of visual acuity, in contrast to progressive recovery of motor functions.

### bMOG EAE induces optic neuritis without RGC death

We sought to determine if optic neuritis and RGC death contribute to visual impairments in bMOG EAE, as reported in MOG_35–55_ EAE [16–20]. FluoroMyelin staining revealed demyelinated areas in optic nerves at day 18 post-bMOG injection (Fig. 1D, E). However, the density of Olig2^+^ oligodendrocytes was not lower in EAE than in intact optic nerves (Fig. 1D, F), suggesting that demyelination occurred without oligodendrocyte death. Glial fibrillary acidic protein (GFAP) and beta 3 tubulin (B3T) staining showed astrogliosis and axonal damage, respectively, in and around demyelinated areas (Additional file 2: Fig. S2). In addition, microglia/macrophages expressing ionized calcium-binding adapter molecule 1 (Iba1) were strongly activated, as indicated by the upregulation of CD68 (Additional file 3: Fig. S3A–D). Importantly, the examination of retinal flatmounts stained for RBPMS, a specific marker of RGCs [40], did not reveal neuronal death at days 18 and 35 (Fig. 1G, H). Together, our results suggest that visual impairment in bMOG EAE results from optic neuritis, characterized by gliosis, demyelination, and axonal loss, but not from RGC death.

### Retinal activity is transiently altered in bMOG EAE

**Fig. 2:**
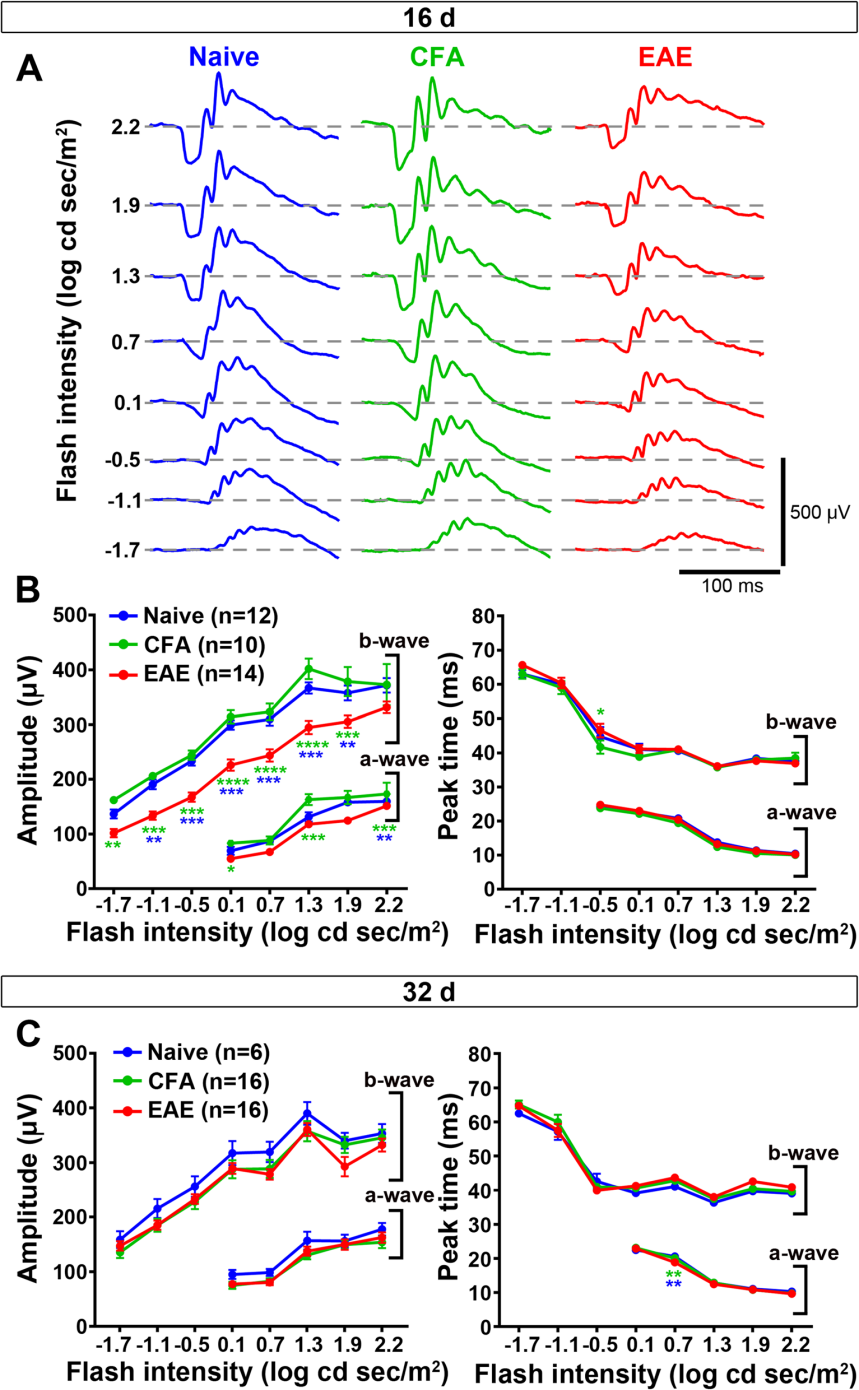
Scotopic electroretinogram response is selectively reduced in the active phase of bMOG EAE. ERGs were recorded at days 16 and 32 after EAE induction. **A** Representative ERG waves are shown on day 16. **B** In EAE mice (*n* = 14), luminance-response curves showed significant reduction in b-wave and a-wave ERG amplitudes compared with CFA (*n* = 10) and naive (*n* = 12) control animals in the active phase of the disease (day 16), as identified in Fig. 1. Peak times were not significantly affected. **C** At 32 days post-bMOG injection, ERG wave amplitudes did not statistically change in EAE mice (*n* = 16) when compared to controls, i.e., CFA-treated (*n* = 16) or naive (*n* = 6) mice. No significant changes were noticed for peak time measurements. Statistics: two-way ANOVA, Tukey post hoc test; *: *P* < 0.05, **: *P* < 0.01, ***: *P* < 0.001, ****: *P* < 0.0001. Green stars indicate statistical significance between EAE and CFA-treated mice. Blue stars indicate statistical significance between EAE and naive mice

**Fig. 3:**
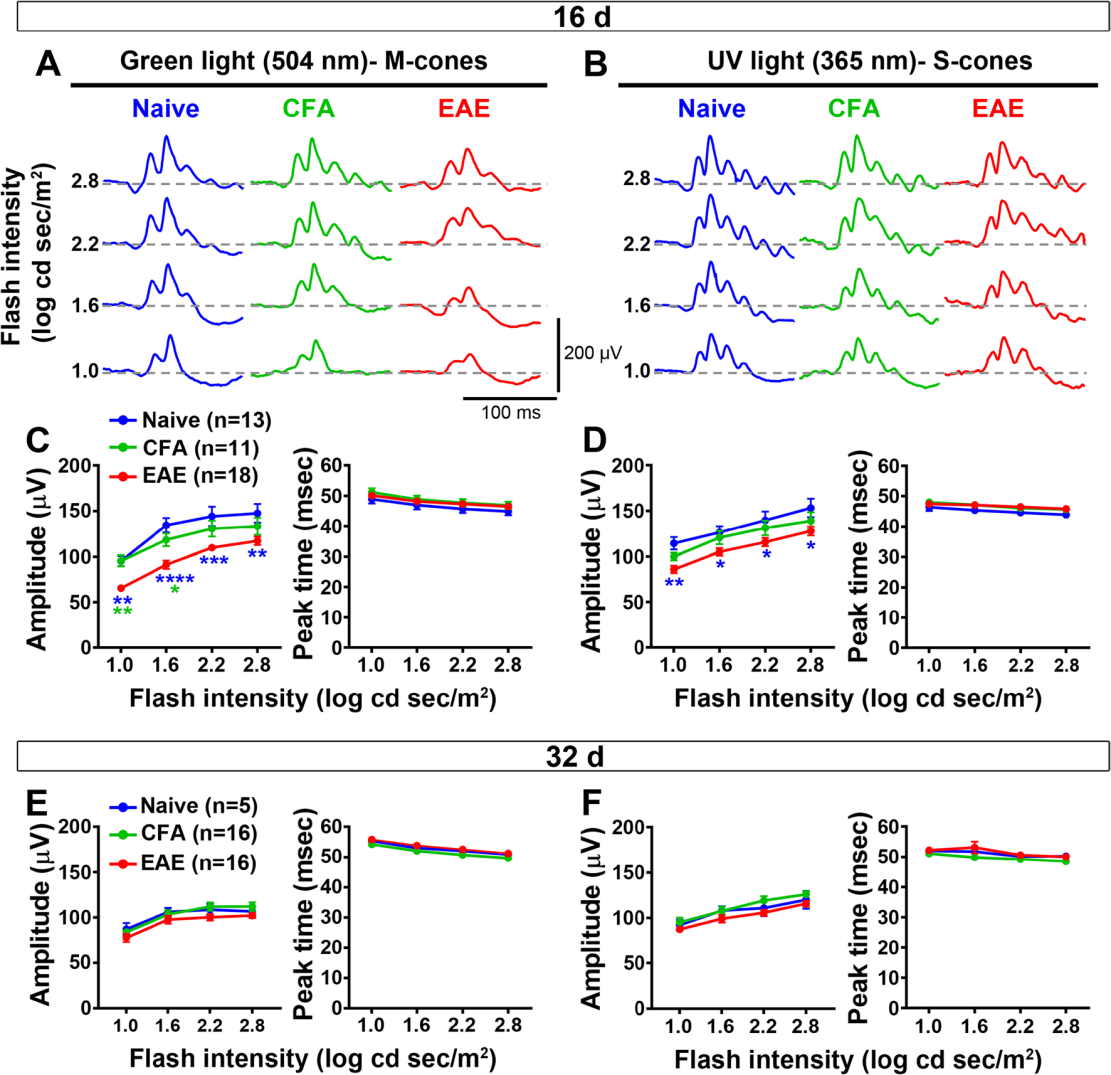
M-cone photopic ERG response is decreased in the active phase of bMOG EAE. **A**, **B** M- and S-cone-mediated photopic ERG responses were, respectively, recorded after flash stimulation at 504 and 365 nm. Representative M-cone-dependent photopic waves showed clear b-wave amplitude reduction at 504-nm stimulation (**A**). Representative S-cone-dependent photopic waves also suggest moderate decrease in retinal activity at 365-nm stimulation (**B**). **C** Quantitative analyses of luminance-response curves revealed strong diminution of M-cone b-wave amplitudes in EAE mice (*n* = 18) compared with CFA (*n* = 11) or naive (*n* = 13) mice. Peak times were not affected by EAE. **D** S-cone-mediated ERG response was only significantly reduced in EAE eyes relative to naive mice, but did not statistically change when compared to CFA-treated mice. Peak times were similar between the groups. **E**, **F** The amplitude and peak time of b-waves induced by M- and S-cone stimulation did not differ between EAE (*n* = 16) and control treatments (CFA, *n* = 16; naive, *n* = 5) on day 32. Statistics: two-way ANOVA, Tukey post hoc test; *: *P* < 0.05, **: *P* < 0.01, ***: *P* < 0.001, ****: *P* < 0.0001. Green stars indicate statistical significance between EAE and CFA-treated mice. Blue stars indicate statistical significance between EAE and naive mice

It has been shown that the unmyelinated retinal cell layers upstream of RGCs are functionally affected in MOG_35–55_ EAE [41]. We therefore evaluated retinal activity by recording ERGs during the peak (day 16) and the chronic phase (day 32) of bMOG EAE. Mice were dark-adapted to obtain scotopic ERGs, from which we measured the amplitude of the a- and b-waves that are, respectively, generated by photoreceptors and inner retinal cells (bipolar and Müller cells). Both waves were weaker in bMOG mice on day 16 (Fig. 2A, B), but went back to normal values on day 32 (Fig. 2C). ERGs were then recorded in daylight to saturate rod photoreceptors and to assess photopic retinal vision, which is mediated by two types of cone photoreceptors with different wavelength sensitivities [42]: M-cones are optimally activated with green light at 504 nm [43], whereas S-cones are more sensitive to UV light at 365 nm [44]. ERGs were thus recorded at both wavelengths (Fig. 3). On day 16, the M-cone-dependent ERG response (b-wave) was significantly decreased in bMOG mice compared to control mice injected with CFA alone or PBS (Fig. 3A, C). The S-cone-dependent response was also reduced in EAE mice, but to a lesser extent, and the difference was statistically significant only compared to the PBS group (Fig. 3B, D). On day 32, no intergroup difference was observed (Fig. 3E, F). Collectively, these results indicate a marked, but reversible decrease in retinal activity during bMOG EAE.

### M-opsin is upregulated in EAE retinae

**Fig. 4:**
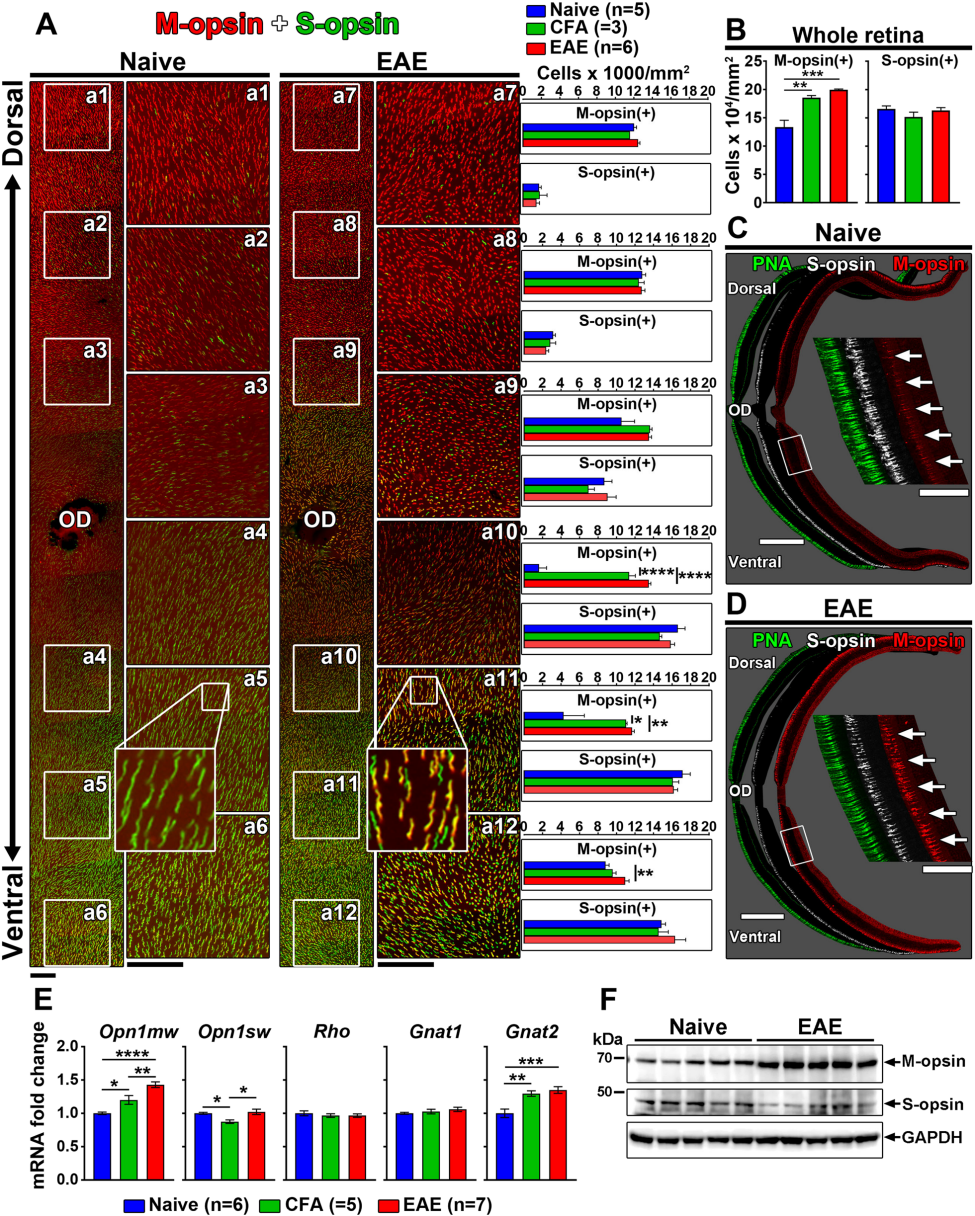
EAE and CFA treatments increase M-opsin expression in cone photoreceptors. **A** Retinal flatmounts were stained for M-opsin and S-opsin by immunofluorescence, 18 days after EAE induction (*n* = 6) or CFA (*n* = 3) or PBS (naïve; *n* = 5) injection. Dorso-ventral gradients of M-opsin and S-opsin could be observed for all groups, with predominant M-opsin staining in the dorsal retina and stronger S-cone signal in the ventral regions. However, in EAE retinae, and to a lower extent in CFA retinae, M-opsin expression appeared relatively higher in ventral cells (Boxes a10 to a12), where S-opsin normally prevails in naive animals (a4–a6). The number of M-opsin-labeled cells was higher in EAE and in CFA ventral retinae than in naive animals. However, this difference was only maintained in the most ventral areas of EAE retinae (a12). **B** Consistently, in the whole retina, the average number of cells expressing M-opsin was increased after EAE induction or CFA injection relative to naive controls, but more strongly in EAE conditions. **C**, **D** Increased expression of M-opsin in the ventral quadrant of EAE retinae could be observed on retinal cross-sections as well. Peanut agglutinin (PNA) was used as a general marker of cone photoreceptor outer segments. **E** On day 18, by RT-qPCR, the mRNA level of *Opn1mw* gene, coding for M-opsin, was higher in EAE retinal lysates (*n* = 7) than in CFA retinal samples (*n* = 5) and naive animals (*n* = 6). Moderate but significant changes in the expression of *Opn1sw*, coding for S-opsin, were found between CFA-treated retinae and other groups. No change was noticed between EAE and naive conditions. Interestingly, the transcript level of *Gnat2*, coding for cone-specific alpha transducing subunit was elevated in EAE/CFA mice, although that of *Gnat1*, coding for rod-specific alpha transducin subunit, did not vary between experimental groups. **F** By Western blotting, M-opsin was upregulated in EAE retinal lysates (*n* = 5) compared with naive lysates (*n* = 5). Scale bars: **A** = 100 µm, **C**, **D** = 400 µm on left, 100 µm (Right close-ups). Statistics: one-way ANOVA, Tukey post hoc test, *: *P* < 0.05, **: *P* < 0.01, ***: *P* < 0.001, ****: *P* < 0.0001. *OD* optic disk

As light activation of M- and S-cones depends on the levels of M-opsin and S-opsin in retinal outer segments [45–47], we wondered whether opsin expression changes could explain photopic ERG function loss in bMOG EAE mice. This possibility was addressed by immunostaining retinal flatmounts for M-opsin and S-opsin (Fig. 4). As expected [31, 48], these opsins showed opposite dorsoventral gradients in normal retinae (Fig. 4A). However, ectopic expression of M-opsin appeared in the ventral cones of the bMOG retinae at day 18, and to a lower extent in those of the CFA controls (Fig. 4A, B). Ventral cones expressing ectopic M-opsin also contained S-opsin (see a11 inset). The number of S-opsin^+^ cones did not decrease in EAE mice, suggesting that M-opsin upregulation did not occur at the expense of S-opsin (Fig. 4A, B). Consistently, ectopic M-opsin expression was obvious in histological sections of bMOG retinae (Fig. 4C, D). In addition, the levels of M-opsin and *Opn1mw* mRNA, encoding M-opsin, were higher in retina lysates from bMOG mice (Fig. 4E, F). The level of *Gnat2* mRNA, encoding a cone-specific transducin subunit, was upregulated in bMOG and CFA mice, whereas that of *Gnat1*, encoding a rod-specific transducin subunit, did not differ (Fig. 4E). These results suggest that M-cones undergo specific gene expression changes associated with decreased scotopic ERG response in bMOG EAE.

### bMOG-induced EAE activates retinal gliosis and inflammation

**Fig. 5:**
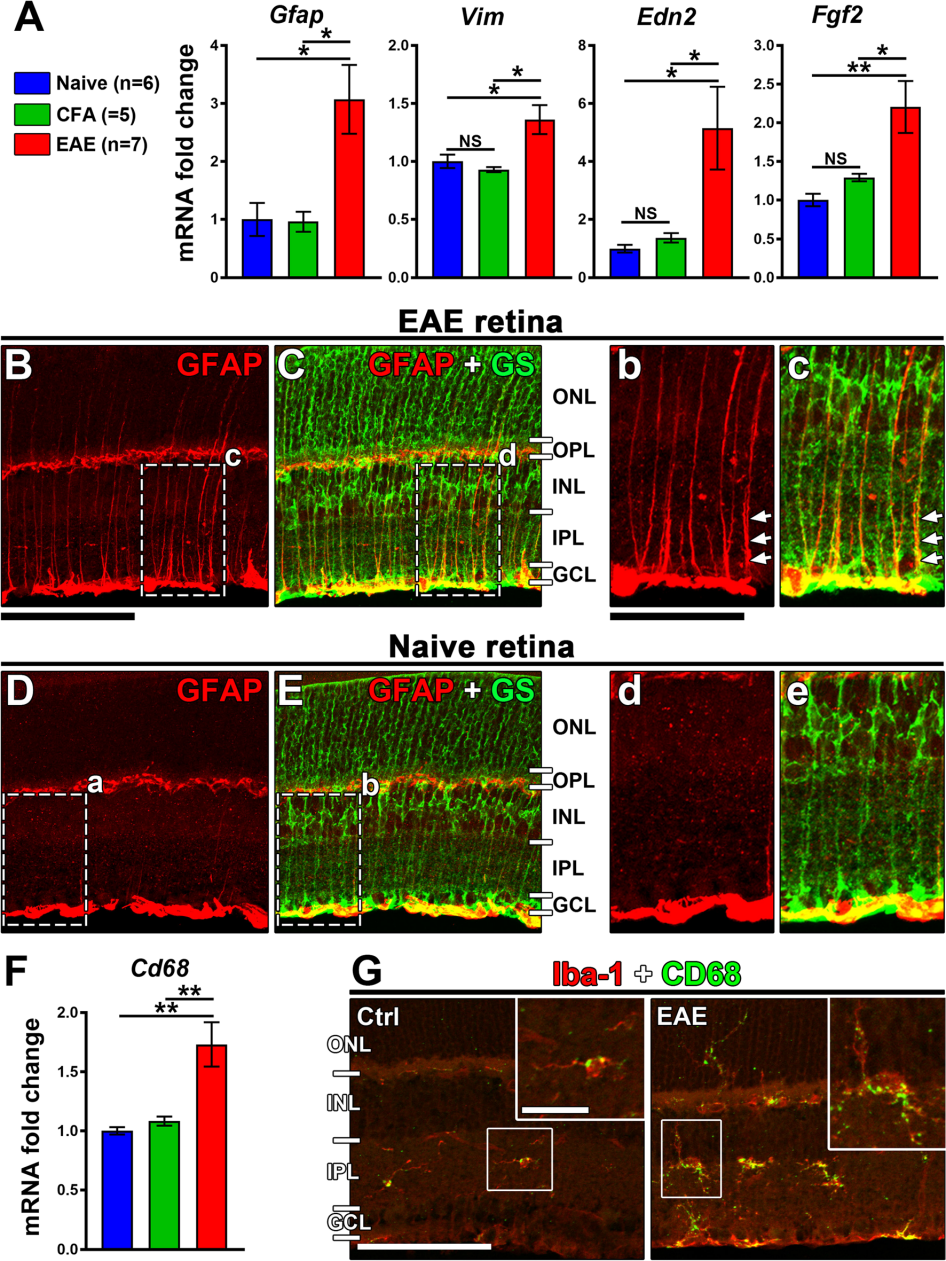
bMOG induces retinal gliosis. **A** Quantification of glial *Gfap*, *Vim* and neuroprotection markers (*Edn2*, *Fgf2*) in retinae by RT-qPCR revealing increased gliosis in EAE. The four markers were significantly higher in EAE (*n* = 7) than in naive (*n* = 6) and CFA (*n* = 5) groups (one-way ANOVA, Tukey post hoc test, *: *P* < 0.05, **: *P* < 0.01, NS = not significant). **B**–**E** Qualitatively, on EAE retinal cryosections, the GFAP signal was stronger in GS^+^ radial Müller cell extensions in comparison to naive mice. At higher magnifications (**b**–**e**), the spread of GFAP was prominent in Müller cell endfeet and in the inner retina. **F** Quantification of *Cd68* in retinal lysates by RT-qPCR suggesting an increased activation of microglia/macrophage in EAE mice (one-way ANOVA, **: *P* < 0.01). **G** Qualitatively, immunofluorescent staining showed higher expression of CD68 in microglial cells identified using the Iba1 marker (red) in EAE retinae. At higher magnification, the dotted distribution of CD68 in microglial cells suggests increased lysosomal activity associated with microglial phagocytosis and activation. Scale bars: **B** = 100 µm, **b** = 50 µm, **G** = 100 µm, close-up in **G** = 25 µm. *ONL* outer nuclear layer, *OPL* outer plexiform layer, *INL* inner nuclear layer, *IPL* inner plexiform layer, *GCL* ganglion cell layer

By RT-qPCR on whole retinal lysates, we measured the expression of different markers, including *Gfap* and *Vim*, which are classical markers of Müller cell gliosis [49], and *Edn2* and *Fgf2,* which encode neuroprotective factors upregulated during retinal injury [50]. All these mRNAs were significantly increased in EAE retinae at day 18 (Fig. 5A). Qualitatively, immunostaining of retinal sections showed higher levels of GFAP in the radial extensions of Müller cells expressing glutamine synthetase (GS) (Fig. 5B–E). Phosphorylation of extracellular signal-regulated kinases 1/2 (Erk1/2), that has been associated with retinal gliosis [40, 51], was also increased in Müller cells (Additional file 4: Fig. S4). Moreover, qualitative observations of CD68 expression on retinal sections suggested microglia/macrophage activation (Fig. 5F, G). Therefore, we conclude that gliosis and inflammation likely contribute to the functional alterations of the retina during bMOG EAE.

### bMOG EAE modifies gene expression in the retina

**Fig. 6:**
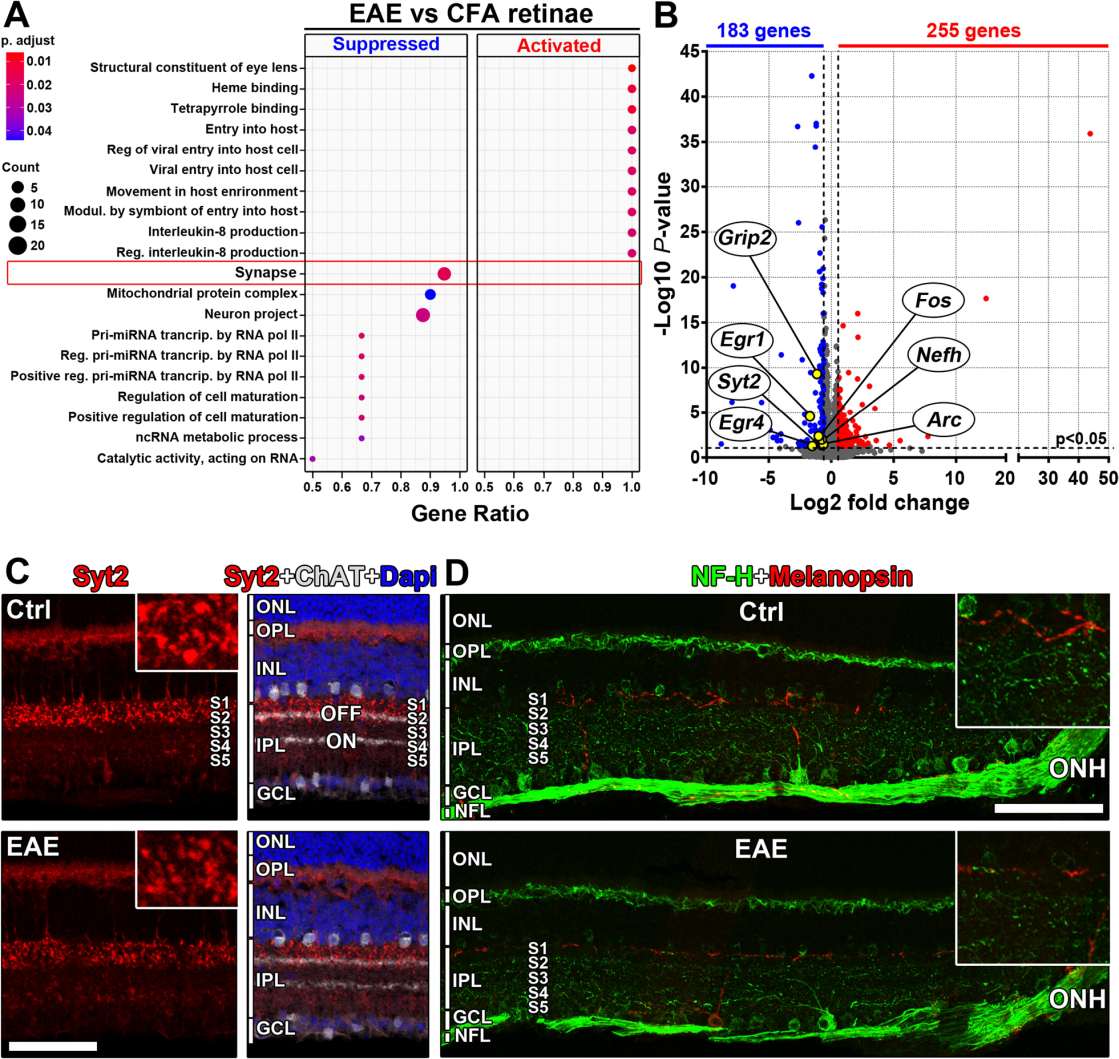
Downregulation of synapse-associated gene expression in EAE. **A** Transcriptomic analysis was carried out by RNAseq. Gene ontology term enrichment analysis revealed the downregulation of genes associated with the synapse (EAE mice, *n* = 8; CFA mice, *n* = 5). **B** On a volcano plot, up- and downregulated genes have been represented. Genes included in the synapse gene ontology term, and depending on neuronal activity, have been highlighted, such as that of *Syt2* and *Nefh*. **C** Synaptotagmin 2, a synaptic protein encoded by *Syt2* and abundant in the terminals of type 2 OFF cone bipolar cells, was decreased in the OFF sublamina 1 (S1). The staining of choline acetyltransferase (ChAT) in starburst amacrine cell dendrites allowed to localize ON and OFF synaptic layers. Dapi nuclear staining was used to observe the outer nuclear layer (ONL), the inner nuclear layer (INL) and the ganglion cell layer (GCL). **D** By immunofluorescence, the signal of Neurofilament H (NF-H), whose level depends on *Nefh* expression, appeared weaker in RGC dendrites (close-up), in the nerve fiber layer (NFL) and in the horizontal cells of the outer plexiform layer (OPL). Melanopsin labeling allowed to visualize the border between the inner plexiform layer (IPL) and the inner nuclear layer (INL). Scale bars: **C** = 50 µm; **D** = 100 µm. *ONH* optic nerve head

To gain further insights into the transcriptomic changes affecting the retina during bMOG EAE, RNAseq was performed on whole retina collected on day 18 (Fig. 6). The expression of genes associated with synaptic activity was particularly decreased, as shown by gene ontology term enrichment analysis (Fig. 6A). This group of genes included *Synaptotagmin 2* (*Syt2)* and *Neurofilament heavy chain* (*Nefh)* (Fig. 6B), which encode neuronal proteins expressed, respectively, in the synaptic terminals of type 2 OFF cone bipolar cells (Fig. 6C) or in horizontal cells and alpha RGCs (Fig. 6D). The downregulation of neuronal activity-dependent genes in the retina therefore adds to the changes contributing to vision loss during bMOG EAE.

## Discussion

This study shows that B cell-dependent EAE causes a persistent decline in visual acuity, which is associated with optic neuritis and demyelination, but not with RGC death. This disease also induced a transient reduction in retinal cell activity, as evidenced by loss of ERG amplitude. In particular, the M-cone pathway appears to be more affected than the S-cone pathway, as shown by photopic ERGs and the upregulation of M-opsin expression. Consequently, we conclude that visual function impairment in B cell-dependent EAE is not only due to inflammation of the optic nerve, but also of the retina. Because the retina lacks myelin, it is most likely affected by a secondary reaction rather than direct attacks by myelin-reactive T cells and antibodies.

### The electroretinogram as a biomarker of disease activity in demyelinating autoimmune diseases

Our ERG results demonstrate that retinal activity is significantly inhibited in bMOG EAE at the peak of disease, a phenomenon that is subsequently reversible. This reversibility likely results from the progressive attenuation of the autoimmune reaction, as suggested by the reduction in motor symptoms. This interpretation is consistent with the inflammatory retinal edema that is observable by optical coherence tomography at the peak of EAE, but not during the late phase [19]. In contrast, other studies in female rats with EAE did not show ERG attenuation [16, 17]. The possibility of observing a decreased ERG response in EAE could therefore depend on experimental conditions, such as the species, sex and antigen.

In human, the limited data available suggest that ERG may be affected in MS as well. Indeed, a reduction of the ERG a-wave and b-wave amplitudes has been reported at the onset of optic neuritis, but not 1 and 6 months later [52]. In a larger cohort, MS patients did not present significant ERG changes when examined long after an acute attack [26]. Taken together, these observations suggest that active inflammatory demyelination can lead to ERG changes in the outer retina. Thus, this study warrants further characterization of ERG changes in patients with autoimmune demyelination and provides a relevant animal model to study the underlying mechanism.

### Potential implication of the M-cone pathway in color vision impairment and in monitoring disease activity

Our observation that the ERG response to green light stimulation is more affected than that elicited by UV light suggests that the M-cone pathway is preferentially affected. Since the mouse retina can discriminate colors in the upper visual field [53–55], this result leads us to believe that color vision is affected in bMOG EAE. In MS patients, loss of color vision (dyschromatopsia) is frequently observed [56, 57] and positively correlates with disease severity [56]. This loss can be explained, at least in part, by RGC death [15, 56–58]. However, it cannot be excluded that cone dysfunction may also contribute, as color vision results from activation of cone photoreceptors [59]. To our knowledge, this possibility has never been studied in MS, perhaps because it is counterintuitive to study a nonmyelinated structure such as the retina. In light of the new results presented here, the role of the M-cone pathway in dyschromatopsia deserves to be examined in patients with autoimmune demyelination by recording chromatic ERGs. Such recording may even be useful for monitoring subclinical disease progression, as dyschromatopsia can be observed in absence of optic neuritis [60, 61].

### Sustained visual function impairments in bMOG-induced EAE

Our OKR results suggest that visual function decrease lasts until 30 days post-bMOG injection, a time point by which the ERG and RGC survival are not affected. Therefore, the loss of vision is not attributable to retinal cell dysfunction and death. Sustained visual function deficits may result from optic nerve inflammation and demyelination, persisting beyond the inflammatory phase of the disease. Myelin is essential for action potential conductance in the optic nerve and for eye-to-brain transmission of visual information. Interestingly, if the integrity of axons is preserved, axonal conductance defects can be overcome in the demyelinated optic nerve by blocking voltage-gated potassium channels with 4-aminopyridine (4-AP) [39, 62]. In a similar fashion, the use of 4-AP in bMOG EAE mice may help clarify the role of axonal demyelination vs axonal lesion in visual acuity loss. Indeed, after bMOG EAE, we observed moderate axonal injury in the optic nerve. In MOG_35–55_-induced EAE, optic nerve axonal damage has been associated with visual function decrease [16, 17, 19]. Axonal lesion permanently prevents the transmission of action potentials from RGCs to neuronal brain targets [63] and leads to retrograde RGC death [64–66]. Although our quantitative analyses of RBPMS^+^ RGCs did not reveal significant cell loss, one cannot exclude the possibility that RGCs are lost at a time point later than 32 days, similarly to what has previously been observed in the classical MOG_35–55_ EAE model [20, 39]. With our current data, we propose that myelin loss and partial axonal injury in bMOG EAE optic nerves are the most plausible causes of sustained OKR decrease.

## Conclusion

In conclusion, bMOG EAE is characterized by visual deficits resulting from: (1) persistent optic nerve inflammation that does not result in RGC death and by (2) reversible retinal impairment. Considering that bMOG EAE is less severe than classical EAE, which results in RGC death, and that it shares features with human diseases such as optic neuritis and MS, we believe that this novel model would be advantageous for testing experimental treatments aiming at protecting axons and oligodendrocytes or to stimulate their regeneration.

## Supplementary Information

Below is the link to the electronic supplementary material.

**Additional file 1: Figure S1.** Motor and visual acuity changes in acute bMOG-induced EAE. The time-course of EAE clinical scores and optomotor visual acuity changes were established in mice. The values shown include those from animals presented in Fig. 1. A) The pattern of EAE clinical score variations was similar to that obtained in mice shown in Fig. 1A. B) The left and right eyes were similarly affected on days 9 and 16. Statistics: two-way ANOVA, Tukey post hoc test, *: *P* < 0.05, **: *P* < 0.01, ***: *P* < 0.001, ****: *P* < 0.0001.

**Additional file 2: Figure S2.** Histological analysis of myelin lesions in EAE optic nerves. Histological analysis of EAE optic nerves revealed clear FluoroMyelin-free areas where astrocytes and axons were labeled with glial fibrillary acidic protein (GFAP) and beta3Tubulin (B3T), respectively. In rostral and caudal regions of EAE optic nerves, the higher density of GFAP^+^ fibers and lesioned B3T^+^ axons appeared in and around demyelinated areas (dotted lines). Zones completely deprived of staining (black holes) systematically appeared in the center of demyelination areas. Scale bar = 100 µm.

**Additional file 3: Figure S3.** Monocyte activation in EAE optic nerves. A) The lysosomal CD68 protein was used as a marker to detect activated microglia/macrophages by immunofluorescence on optic nerve sections. The CD68 fluorescent signal was strong throughout the optic nerve of EAE mice compared with naive controls. B) High resolution pictures acquired by confocal microscopy showing coexpression of CD68 with the monocyte marker Iba1. C) Quantitative measurements showed a significant increase in the optic nerve surface positive for CD68 in EAE mice relative to naive animals (Unpaired *t*-test, **: *P* < 0.01). D) Interestingly, CD68 staining was stronger in the rostral than in the caudal part of EAE optic nerves (Paired *t*-test: *: *P* < 0.05). Scale bars: *A* = 200 µm, *B* = 50 µm.

**Additional file 4: Figure S4.** Erk1/2 phosphorylation is upregulated in EAE Müller cells. Immunofluorescence on retinal cryosections showed that Erk1/2 was more phosphorylated in EAE than in naive mice. The increased signal of P.Erk1/2 was colocalized with glutamine synthetase (GS), a specific marker of Müller glia. Retinal cell layers stained with DAPI allowed to observe the distribution of P.Erk1/2 in the radial extensions of Müller cells and in their cell body localized in the middle of the inner plexiform layer (IPL). Scale bars: A–D = 100 µm, C, D = 20 µm. *ONL* outer nuclear layer, *OPL* outer plexiform layer, *INL* inner nuclear layer, *GCL* ganglion cell layer.

## Data Availability

The datasets used and/or analyzed during the current study are available from the corresponding author on reasonable request.

## Abbreviations

B3T: Beta 3 tubulin
CD68: Cluster of differentiation 68
CFA: Complete Freund’s adjuvant
CNS: Central nervous system
EAE: Experimental autoimmune encephalomyelitis
Edn2: Endothelin 2
Erk1/2: Extracellular signal-regulated kinases ½
ERG: Electroretinogram
Fgf2: Fibroblast growth factor 2
GFAP: Glial fibrillary acidic protein
Gnat1: G Protein Subunit Alpha Transducin 1
Gnat2: G Protein Subunit Alpha Transducin 2
GS: Glutamine synthetase
Iba1: Ionized calcium-binding adaptor molecule 1
MOG: Myelin oligodendrocyte glycoprotein
MS: Multiple sclerosis
Nefh: Neurofilament heavy chain
OKR: Optokinetic reflex
olig2: Oligodendrocyte transcription factor 2
Opn1mw: Opsin 1, medium wave sensitive
Opn1sw: Opsin 1, short wave sensitive
PBS: Phosphate buffer saline
PFA: Paraformaldehyde
RBPMS: RNA binding protein with multiple splicing
RGC: Retinal ganglion cell
Syt2: Synaptotagmin 2
Vim: Vimentin
