# Supplementary figures and images for: B cell-dependent EAE induces visual deficits in the mouse with similarities to human autoimmune demyelinating diseases
Source: J Neuroinflammation. 2022 Feb 23;19:54. doi: 10.1186/s12974-022-02416-y (PMC8867627; doi:10.1186/s12974-022-02416-y)

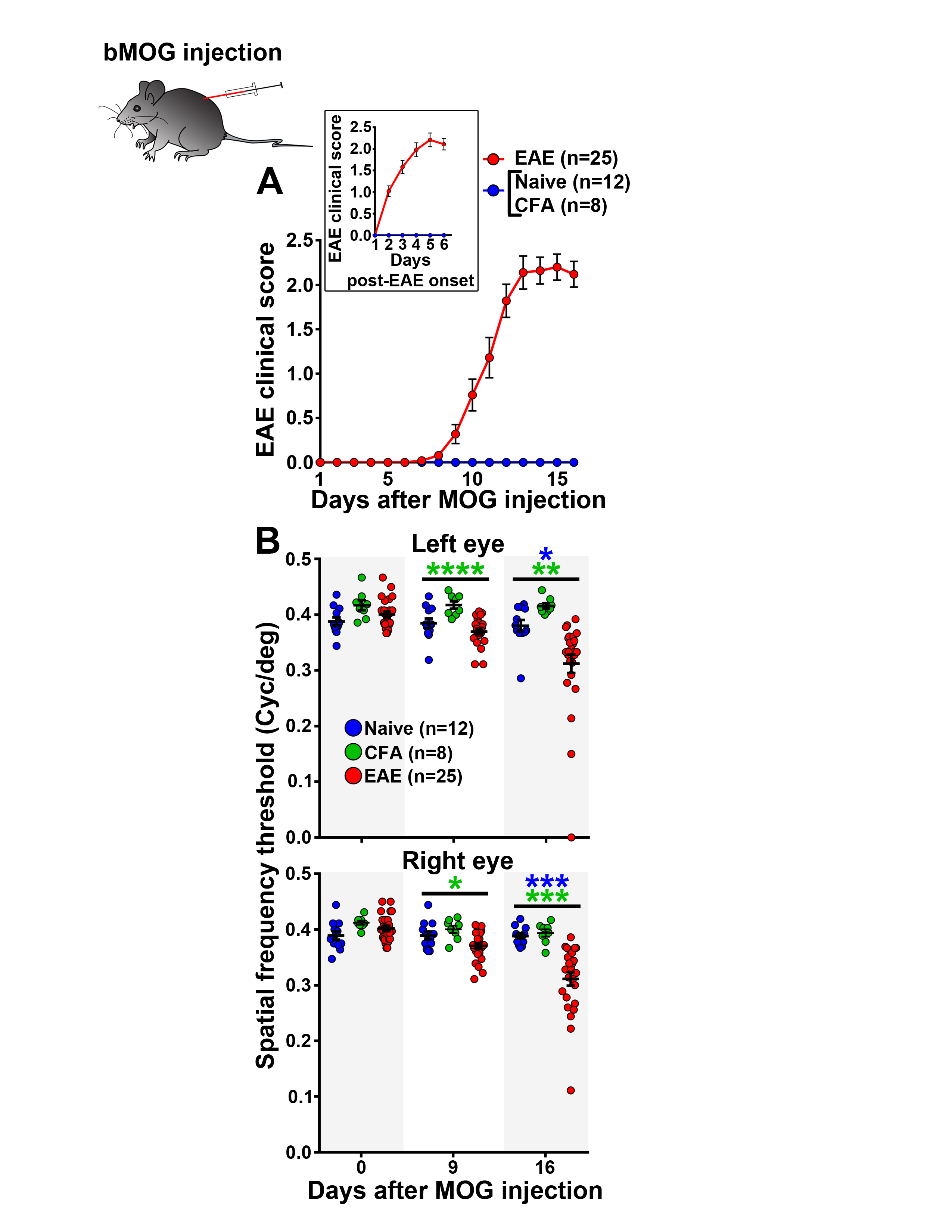

Supplement: Supplementary file 1 — Additional file 1: Figure S1. Motor and visual acuity changes in acute bMOG-induced EAE. The time-course of EAE clinical scores and optomotor visual acuity changes were established in mice. The values shown include those from animals presented in Fig. 1. A) The pattern of EAE clinical score variations was similar to that obtained in mice shown in Fig. 1A. B) The left and right eyes were similarly affected on days 9 and 16. Statistics: two-way ANOVA, Tukey post hoc test, *: P < 0.05, **: P < 0.01, ***: P < 0.001, ****: P < 0.0001. [file 12974_2022_2416_MOESM1_ESM.tif]

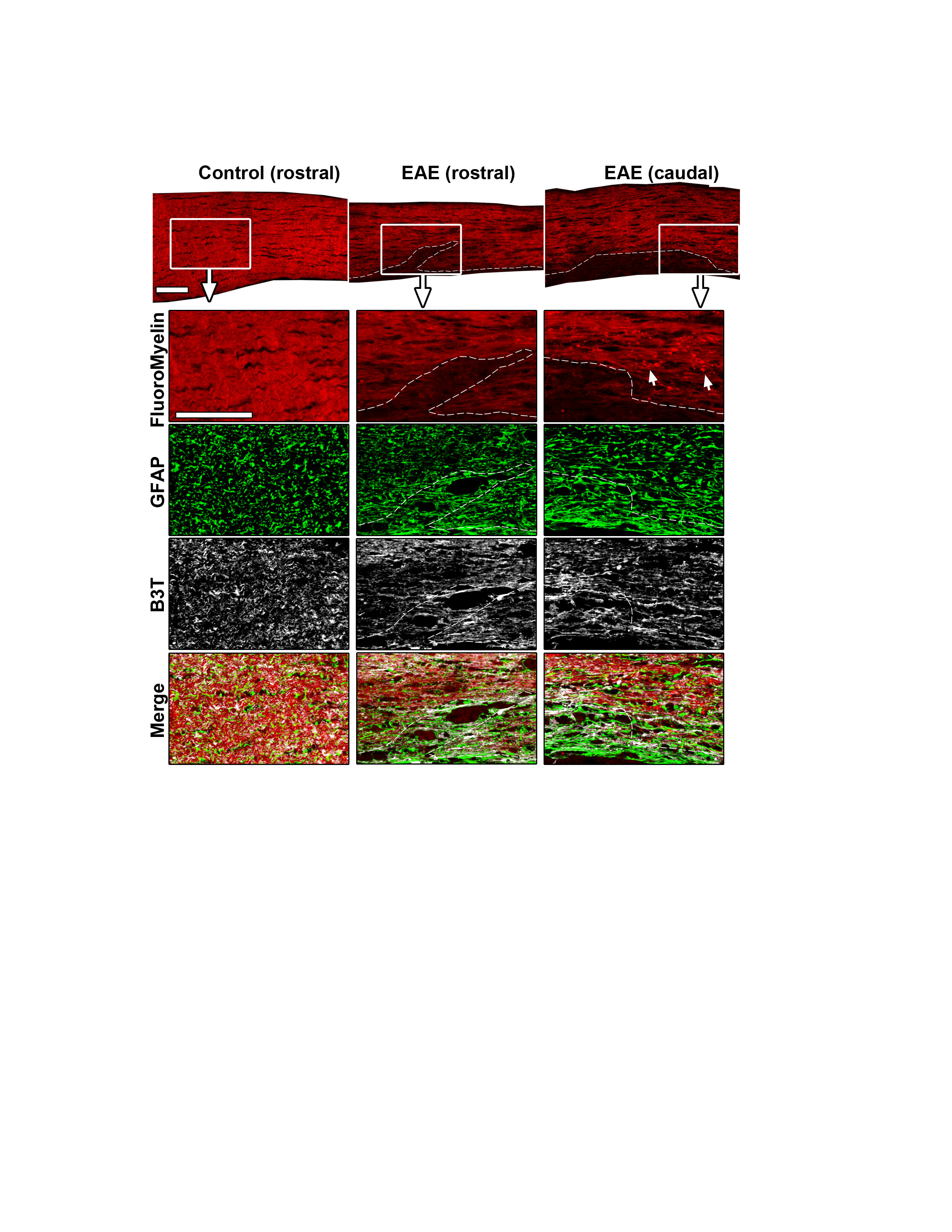

Supplement: Supplementary file 2 — Additional file 2: Figure S2. Histological analysis of myelin lesions in EAE optic nerves. Histological analysis of EAE optic nerves revealed clear FluoroMyelin-free areas where astrocytes and axons were labeled with glial fibrillary acidic protein (GFAP) and beta3Tubulin (B3T), respectively. In rostral and caudal regions of EAE optic nerves, the higher density of GFAP+ fibers and lesioned B3T+ axons appeared in and around demyelinated areas (dotted lines). Zones completely deprived of staining (black holes) systematically appeared in the center of demyelination areas. Scale bar = 100 µm. [file 12974_2022_2416_MOESM2_ESM.tif]

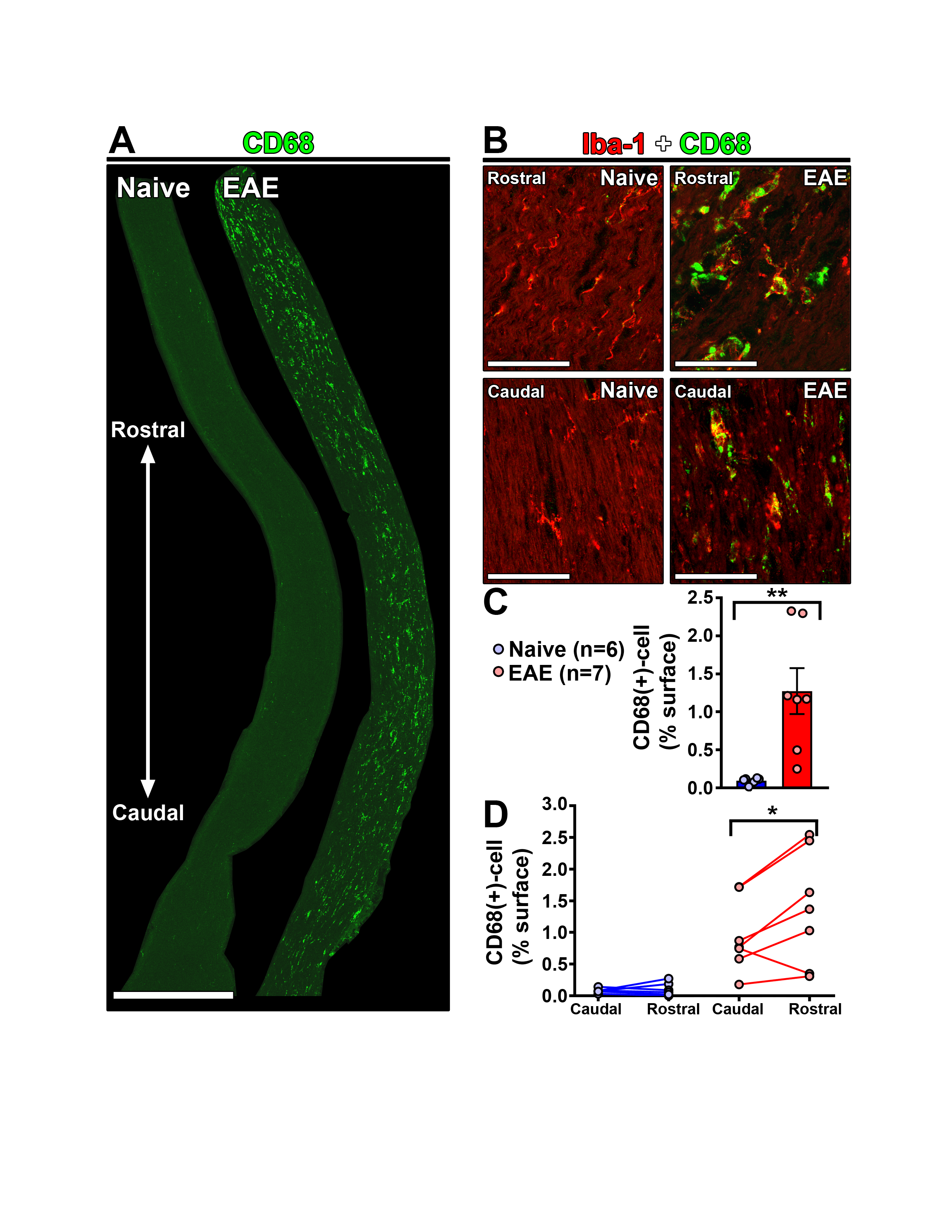

Supplement: Supplementary file 3 — Additional file 3: Figure S3. Monocyte activation in EAE optic nerves. A) The lysosomal CD68 protein was used as a marker to detect activated microglia/macrophages by immunofluorescence on optic nerve sections. The CD68 fluorescent signal was strong throughout the optic nerve of EAE mice compared with naive controls. B) High resolution pictures acquired by confocal microscopy showing coexpression of CD68 with the monocyte marker Iba1. C) Quantitative measurements showed a significant increase in the optic nerve surface positive for CD68 in EAE mice relative to naive animals (Unpaired t-test, **: P < 0.01). D) Interestingly, CD68 staining was stronger in the rostral than in the caudal part of EAE optic nerves (Paired t-test: *: P < 0.05). Scale bars: A = 200 µm, B = 50 µm. [file 12974_2022_2416_MOESM3_ESM.tif]

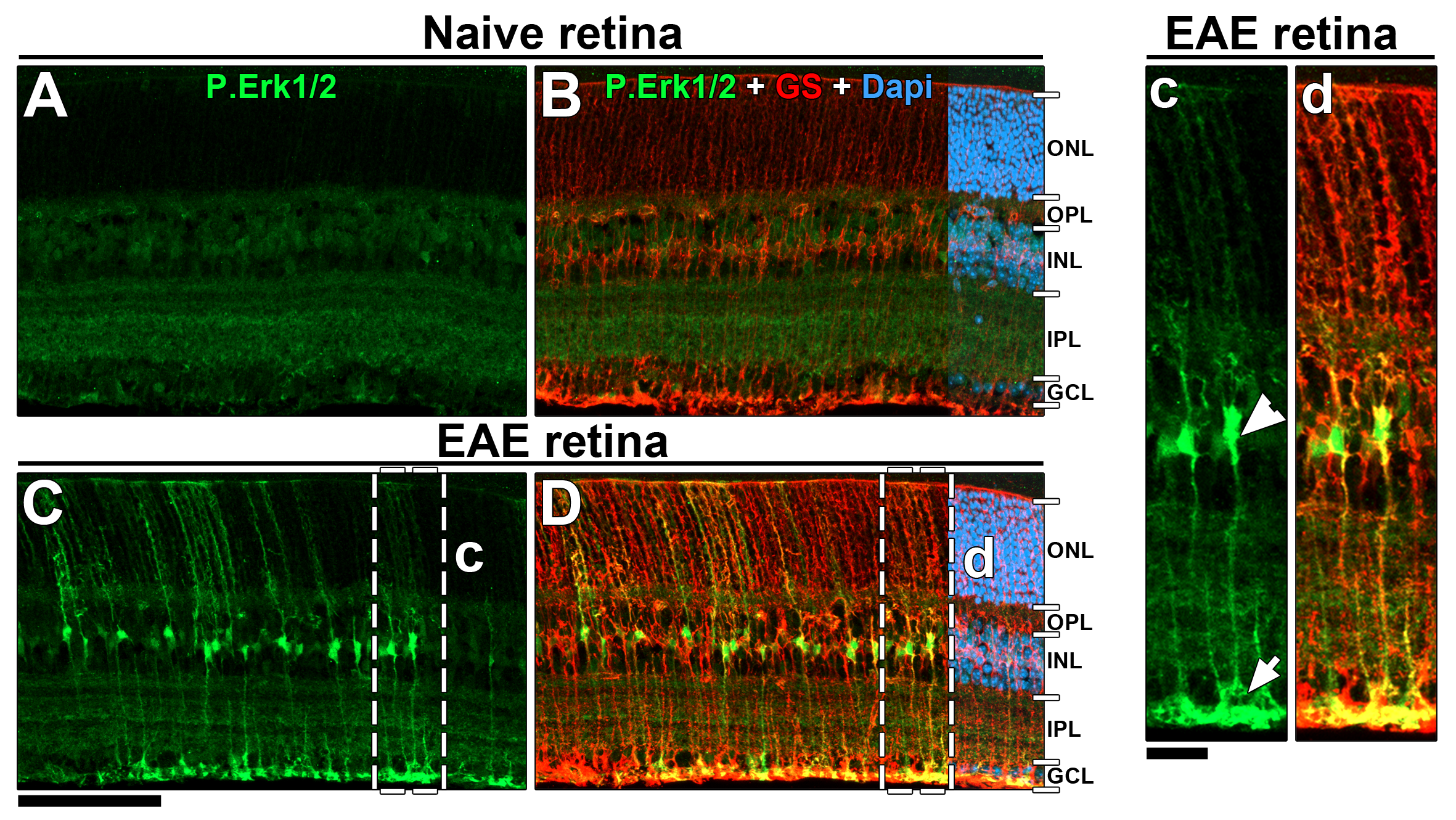

Supplement: Supplementary file 4 — Additional file 4: Figure S4. Erk1/2 phosphorylation is upregulated in EAE Müller cells. Immunofluorescence on retinal cryosections showed that Erk1/2 was more phosphorylated in EAE than in naive mice. The increased signal of P.Erk1/2 was colocalized with glutamine synthetase (GS), a specific marker of Müller glia. Retinal cell layers stained with DAPI allowed to observe the distribution of P.Erk1/2 in the radial extensions of Müller cells and in their cell body localized in the middle of the inner plexiform layer (IPL). Scale bars: A–D = 100 µm, C, D = 20 µm. ONL outer nuclear layer, OPL outer plexiform layer, INL inner nuclear layer, GCL ganglion cell layer. [file 12974_2022_2416_MOESM4_ESM.tif]
